# A Systematic Review of Digital Deformities in Pediatric Patients and Their Podiatric Treatments

**DOI:** 10.3390/children12111461

**Published:** 2025-10-28

**Authors:** Maria Jose Chiva Miralles, Raquel Gil Vargas, Adrian Jorda Vallés, Lucia Carbonell Jose, María Benimeli-Fenollar, Carmen García-Gomariz, José-María Blasco

**Affiliations:** 1 Nursing Department, University of Valencia, 46010 València, Spain; adrian@uv.es (A.J.V.); lucia.carbonell@uv.es (L.C.J.); maria.benimeli@uv.es (M.B.-F.); carmen.garcia-gomariz@uv.es (C.G.-G.); 2 Ankle and Foot Reserch advances Group—GAITP, Department of Nursing, University of Valencia, 46010 València, Spain; 3 Degree in Podiatry, University of Valencia, 46010 València, Spain; ragilvar@alumni.uv.es; 4 Department of Physiotherapy, University of Valencia, Calle Gascó Oliag 5, 46010 València, Spain; jose.maria.blasco@uv.es

**Keywords:** digital deformities, pediatrics, foot

## Abstract

Background and objectives: Digital deformities in pediatrics are one of the reasons why parents go to podiatry clinics with their children. There is a great diversity of digital alterations in the feet; the origin of the vast majority is genetic. Due to this diversity, different treatments are offered depending on deformity and age, ranging from monitoring progress and changing footwear to deformities that are directly evaluated for surgery. The main objective of this study is to describe and identify the different podiatric digital deformities present in pediatric patients, as well as the most commonly used treatments depending on the deformity. Methods: This study is a bibliographic review that has been carried out after using different search equations and applying a series of inclusion and exclusion criteria, obtaining a total of 10 articles (N = 10). Results: A total of 50% of the studies belong to samples where the authors affirm that surgical treatment is the most appropriate in deformities such as polydactyly and macrodactyly, treated in the first months of life. Half of the studies reviewed were clinical studies with a sample, and the other half were bibliographic reviews. Conclusions: Clinodactyly, syndactyly, and macrodactyly are the most frequent deformities, with the first, second, and third toes being the most affected.

## 1. Introduction

Digital deformities in pediatric patients, from 0 to 15 years old, represent a common problem and have become an issue that worries parents. Because of this, it is important to know the morphology and functionality of the foot [1,2].

Most of these alterations are pathologies that can be observed in one of the patient’s immediate family members. They are usually presented from birth and are generally not limited during childhood, although they can become a rigid and painful deformity upon reaching adolescence or adulthood [3].

### Digital Deformities

Polydactyly: The child has supernumerary toes, that is, more than five. It is one of the most frequent digital anomalies, and the incidence is 1.7/1000 live newborns with the same prevalence in both sexes [4,5,6]. It is believed that the main cause is genetic, and it may be a complex genetic syndrome or occur in isolation. It generally involves the border toes, such as the hallux and the fifth [2,5]. The Temtamy and McKusick classification indicates that polydactyly is divided into three groups: preaxial (medial radius; 15%), postaxial (lateral radius; 79%), and central (central rays; 6%) [6]. Treatment varies depending on the degree of affection, whether it is only the distal phalanx, and whether the entire finger is involved or only the soft tissues [5,7,8,9,10]. The goal of surgical reconstruction is to achieve a stable, mobile, pain-free foot with five cosmetically appealing toes that allow for normal footwear and painless ambulation [11].

Oligodactyly: It is a genetic digital deformity, in which the child is born with the absence of one of the toes. If there is no family history, it may also be associated with other diseases, such as Roberts syndrome [2]. The remaining toes may be perfectly functional, so it is usually well tolerated and does not require treatment. If there is an absence of more than two toes with their corresponding metatarsal areas, biomechanical alterations may appear [5,7]. Usually, it is associated with fibular insufficiency or cleft foot syndrome.

Syndactyly: It consists of membranous fusion of the toes; it does not usually affect their function and is usually more of an esthetic problem. It is believed that its origin is genetic, with an autosomal dominant inheritance pattern [2,5,8] and occurs in approximately one in every 2000 to 2500 newborns. Its occurrence in males is twice as common as in females, and mothers aged 40 years or older are more likely to produce children with inborn limb deformities compared to mothers who are 30 years of age or younger. It is more common between the second and third fingers, that is, in the second interdigital space [5,11,12]. Two forms are distinguished where one of them sees a lax membrane (lax membranous syndactyly) join the toes from the commissure to the end and another in which the union of the toes is carried out by the skeleton itself (closed syndactyly). Syndactyls in which only the soft tissues are affected do not usually require treatment, while those in which the phalanges are affected do need it [2,11,13].

Clinodactyly: It is defined as the abnormal deviation in abduction or adduction of one or more toes and is the most common deformity in the pediatric population [2,14]. This deviation is observed more clearly from the transverse and frontal plane. This deformity includes all those that consist of the deviation of a toe, such as quintus varus, hallux varus, and hallux valgus [12].

1. Quintus varus. Adduction of the fifth toe with some external rotation, the metatarsophalangeal joint is dorsiflexed, and the nail plate of this toe is usually smaller than normal. Furthermore, when deviated in varus, the fifth toe may be positioned above the fourth (supraductus) or below (infraductus). It frequently occurs bilaterally and is evenly distributed among boys and girls. This deviation causes the fifth metatarsal head to be more prominent on the outside of the foot, giving rise to what is known as a “tailor’s bunion.” [5].
  Regarding treatment, in the case of newborns, it can be corrected with passive stretching, silicone orthoses, and/or splinting bandages. However, if the child begins to walk without correcting the deformity, it may become rigid or structured and painful. Correction is necessary, and treatment may be surgical [2,5].
2. Hallux varus. It consists of the deviation of the big toe toward the medial line of the body, that is, toward the inner part of the foot. It is a rare deformity. Conservative treatment is insufficient, so it often fails and requires surgery [2].
3. Hallux valgus. The first metatarsal is deviated medially, and the first toe is deviated laterally, so the first metatarsophalangeal joint is prominent, presenting a protuberance known as a bunion. This deformity is commonly called a “bunion”. Between 5 and 10% of children under 14 years of age suffer from it. It is a hereditary condition and rarely symptomatic in this age group, but it is important to consider that it is progressive in children. There are also intrinsic factors in the development of this deformity, such as ligament hypermobility, pronation, or the length of the first metatarsal. In all cases, treatment should begin conservatively. The first therapeutic option is usually the use of an orthosis or splint; in cases of pain, surgical treatment should be delayed as long as possible. Unlike in adults, surgical treatment in children is indicated by pain, not by the degree of deformity. Currently, the development of percutaneous techniques has significantly shortened both surgical time and recovery time for various forefoot deformities [2,11].

Campodactyly: This deviation, unlike clinodactyly, is seen more clearly from the sagittal plane. It is usually bilateral and the most affected fusion of the toes are the second to fifth. These four toes are triphalangeal, unlike the hallux, which consists of two phalanges, and are made up of several joints. If it affects the first toe, it is called hallux flexus [2,15].

There are three types, depending on the joint, that are affected:

1. Claw toe: It is characterized by hyperextension or dorsiflexion of the metatarsophalangeal joint and plantar flexion of the proximal and distal interphalangeal joint [13,14].
2. Hammertoe: The toe presents hyperextension or dorsiflexion of the metatarsophalangeal joint, plantar flexion of the proximal interphalangeal joint, and hyperextension of the distal interphalangeal joint [11,13,14].
3. Mallet toe: The metatarsophalangeal and distal interphalangeal joints are in a normal position, in extension, while the distal interphalangeal joint is in plantar flexion [11,13,14]. If the deformity is flexible, that is, if it can be reduced and placed in its natural shape, conservative treatment is chosen, such as silicone orthoses and stretching of the flexor and extensor muscles. On the contrary, if the toe remains rigid when trying to manipulate it, they will resort to surgery [11].

Macrodactyly: It is a congenital anomaly of unknown origin that consists of hyperplasia of the phalanges and soft tissues of one or more toes. It can occur isolated or accompanied by different anomalies such as hemangiomatosis, neurofibromatosis type 1, arteriovenous malformations, or congenital lymphedema. It has a prevalence of 0.08 per 10,000 newborns [2,15]. Bilateral affection is more common than unilateral affection and treatment must be personalized, taking into account the age of the child, the degree of hyperplasia and the affected fingers. The different treatments range from soft tissue resection, epiphysiodesis, arthrodesis, and osteotomies to total or partial amputation of a radius [15].

Microdactyly: It consists of a hypoplasia of the phalanges, with its corresponding metatarsal either being hypoplastic or not. This fact is typical of the fourth metatarsal [2]. Furthermore, it is usually an isolated deformity that does not usually require treatment, since it does not usually cause disability [2].

Hyperphalangism: It is exceptionally seen in consultations that, on some occasions, the first toe (hallux) with 3 phalanges can be observed. As it is triphalangeal instead of biphalangeal, it is longer and, therefore, the treatment consists of reducing the finger with a normal surgical operation, thus converting it into biphalangeal [2].

Brachydactyly: This deformity implies having one or more toes shorter than normal. It corresponds to a premature epiphysiodesis of the metatarsal, with the shortening of the toe appearing during growth [16,17]. It occurs in 1 in every 2500 births and is generally discovered at birth, with a proportion of 15–25 girls for every boy. It usually occurs in isolation, although the authors also relate it to other syndromes such as achondroplasia and Down syndrome [16,17]. It does not cause pain and there is no specific treatment, since it is a purely esthetic problem and does not cause difficulties when walking [16,17].

Amniotic Bands:

This is a rare condition that includes a group of congenital malformations that often affect distal parts of the body, such as the limbs and head. These malformations appear to result from the constriction or compression of normal fetal structures: constriction rings, amputations, pseudosyndactyly, and exencephaly [5,17].

Limb involvement is the most common. The exact incidence of amniotic band syndrome is difficult to determine; it is estimated at approximately 1/10,000 live births, but is likely higher due to the existence of early, lethal forms. According to most publications, there is no predisposition based on sex or ethnicity [5].

Among the different clinical features of this condition, pseudosyndactyly or acrosyndactyly is noteworthy. The skin fusion is only distal and always spares the proximal part of the web, giving the appearance of fenestrated syndactyly. Therefore, it is very different from genetic syndactyly, which always affects the proximal web.

Treatment should begin as early as possible, usually between 6 months and 1 year of age, to prevent residual deformities such as clinodactyly or camptodactyly, allowing for proper finger growth. Treatment is surgical.

Amniotic bands are caused by damage to a part of the placenta, specifically the amniotic membrane, and can compress parts of the fetus. Simple bands cause inflammation of the distal part of the toe with accompanying lymphedema; this is primarily a cosmetic problem and usually does not require treatment. In contrast, complex bands can lead to complete amputation, especially those that cause neurovascular compromise; these must be surgically released.

## 2. Methods

### 2.1. Study Design

A systematic review of the literature was conducted in accordance with the File S1: PRISMA 2020 guidelines [18]. The review protocol was not registered in any database (e.g., PROSPERO).

### 2.2. Objectives

General objective: To describe and identify the different podiatric digital deformities present in pediatric patients.

Specific objectives:

To analyze possible treatments depending on the deformity.

To determine the importance of early detection.

Information sources and search strategy

The search was carried out in PubMed and Scopus in March 2024. Different combinations of keywords related to pediatric digital deformities were used. The following search equations were applied:

“Digital deformities” AND “Children” AND “Foot” AND “Toes” (98 results).

“Digital pathology” AND “Children” AND “Foot” (94 results).

“Polydactyly” AND “Children” AND “Foot” (184 results).

“Clinodactyly” AND “Children” AND “Foot” (45 results).

“Digital deformities” AND “Pediatric” AND “Foot” AND “Toes” (22 results).

**Figure 1:**
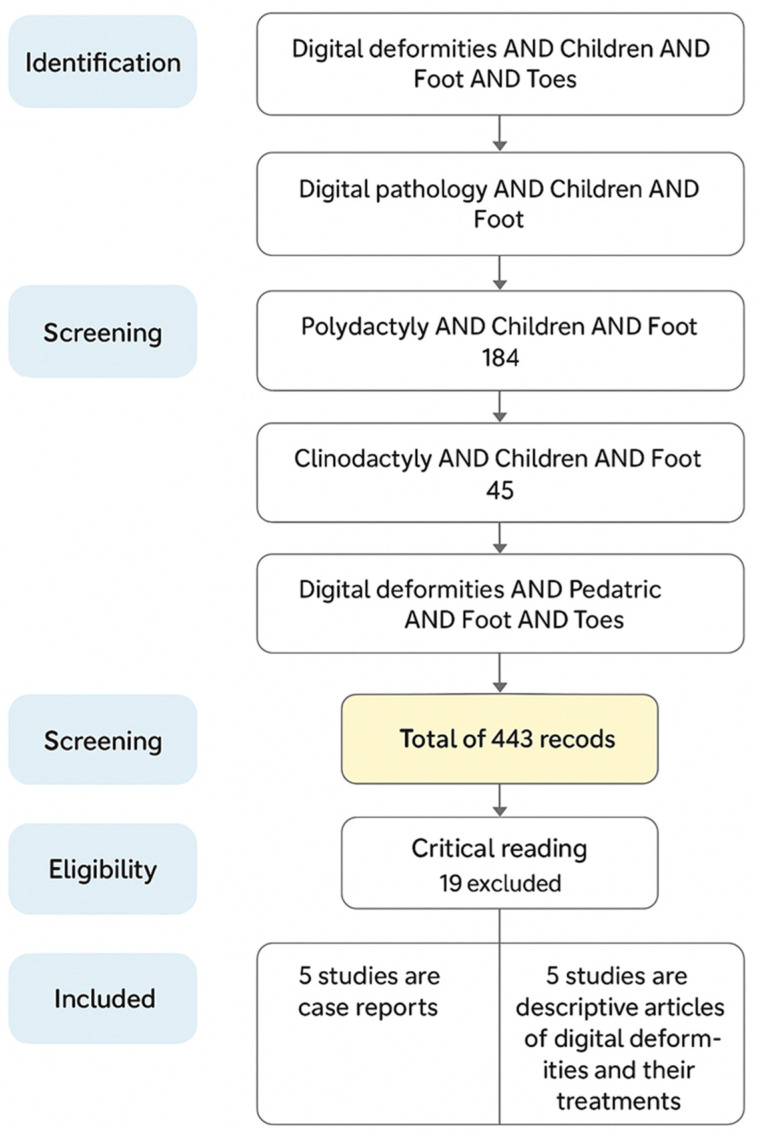
PRISMA flowchart demonstrating the process of paper identification for review.

In total, the initial search identified 443 articles. After applying the filters Free full text and last 13 years, the number of results was reduced to 29 articles (2, 4, 19, 2, and 2 articles from each search, respectively). A critical full-text reading was then conducted, and 19 articles were excluded for not meeting the inclusion and exclusion criteria. Finally, 10 articles were included in the review. The selection process is summarized in the PRISMA flow diagram (Figure 1).

Inclusion criteria:

- Pediatric patients aged 0–15 years.
- Studies focused on digital deformities of the foot.
- Full-text availability.
- Publications from the last 13 years (2011–2024).
- Articles published in English or Spanish.

Exclusion criteria:

- Studies involving adult patients.
- Digital deformities of the hands.
- Studies without access to full text.
- Publications prior to 2010.
- Articles published in languages other than English or Spanish.

Study selection process

- Article selection was independently performed by four reviewers, and discrepancies were resolved by consensus.

### 2.3. Data Extraction and Analysis

Data extraction was cross-checked by the reviewers to ensure consistency. Due to the heterogeneity of the included studies, they were classified into two groups:

Studies with a sample: data extracted included year of publication, sample size, age range, pathology described, affected toes, and treatment applied.

Bibliographic or review studies: data extracted included year of publication, pathology, and recommended treatment.

### 2.4. Quality Assessment

The development of this study, the CASPe method (Critical Appraisal Skills Program, Spain) was employed as a structured tool for the critical appraisal and analysis of scientific evidence. This approach was fundamental in assessing the methodological quality, validity, and applicability of the selected articles, ensuring that they met the necessary criteria for inclusion in the research. By applying the CASPe method, a rigorous and systematic evaluation of each article was conducted, guaranteeing that the studies considered were relevant, reliable, and appropriate to the objectives of this work. The evaluation process considered case report articles and review articles separately, and all were determined to be of sufficient quality for inclusion in the study (see File S2).

### 2.5. Synthesis of Results

Given the heterogeneity of the study designs and outcomes, no meta-analysis was conducted. Instead, a narrative synthesis of the findings was performed.

## 3. Results

This systemic review is composed of a total of ten studies that meet the aforementioned inclusion and exclusion criteria. Therefore, they can be divided into two large, well-differentiated groups: studies that present a sample or a specific clinical case, and those that define different digital deformities with their possible therapeutic options. Those that are studies with a sample or with clinical cases are a total of five articles, and the variables to be studied are as follows: year of publication, sample size, age range, pathology, affected toes and treatment applied.

There are also five bibliographic studies that define various deformities and their treatments. In this case, its variables are as follows: year of publication, pathology and treatment of choice. Table 1 and Table 2 analyze the articles used in the bibliographic study. The variables are made based on the structure and content of the selected articles. Furthermore, the age of the patients used as a sample of the studies ranges from 0 to 15 years, thus covering the entire age range belonging to the pediatric population.

Regarding the results obtained from the case study analysis, these include articles presenting the results of a single case or even other studies that include a sample of 46 patients. The ages covered in these studies range from 0 months to 15 years.

Regarding the different pathologies developed in the studies, the treatment results were as follows:

The deformities with published clinical cases have been clinodactyly, macrodactyly, and one case of hexadactyly, with the second and third toes being the most affected. Regarding the treatments presented, almost all authors, except Chang et al., refer to surgical treatments. Regarding clinodactyly, this author, however, refers to the passage of time and wide shoes as treatments, and if the condition has not been resolved by age 4, flexor tenotomy is considered.

In contrast, the remaining authors, with all the pathologies described—macrodactyly, hexadactyly, and central polydactyly—refer solely and exclusively to surgical treatments as the first and only option.

Something that the 5 studies belonging to this group of articles also have in common is that they all highlight the importance of a careful and exhaustive preoperative evaluation—a good clinical and radiological evaluation that justifies the intervention. In addition to explaining how the postoperative results have been, if the treatment applied has been effective or if any type of subsequent complication has occurred. Throughout this review, it is stated that the most common podiatric digital deformity in the pediatric population is clinodactyly, in the study by Chang et al. This information is reaffirmed, since it is the deformity that most children in the study present [11].

Of the various conditions treated, the only one requiring surgical treatment as a first option is amniotic bands. For all other conditions, including Hallux valgus, Hallux interphalangeal, as well as supraductus and infraductus, the authors (Riera Campillo, M. and Rampal V, Giuliano F.) recommend initially assessing the patient’s progress and applying conservative treatments. Montón Álvaréz J.L even recommends waiting until the age of 5 to make surgical decisions.

In his study on hallux valgus, Arroyave del Río IV refers to orthoses, changes in footwear, and plantar supports, but does not consider surgery until the bone tissue has fully grown. Unlike Montón Alvaréz, who suggests waiting until the age of 5, which is the age at which the bone is not yet fully formed.

The description and identification of these deformities is very useful in detecting them as early as possible. The studies reviewed show that the age factor is very important. In some cases, early detection becomes more important, as is the case with clinodactylies and campodactylies. If the boy or girl begins to wander without being detected, it can convert a flexible deformity into a rigid one. Delay in detection can also impact treatment, which differs depending on the deformity studied. However, for all deformities, conservative treatment, whether with orthoses, plantar supports, or appropriate footwear, can improve the symptoms and the patient’s life, even if it does not improve the deformity. As stated by Tatsuya et al., regarding syndactyly surgeries, one of the most frequent complications is the formation of keloids, a complication that is both serious and bothersome for patients.

## 4. Discussion

To discuss the ten selected articles, the aforementioned division is followed.

### 4.1. Studies with Sample

Before revealing the results and the therapeutic strategy being carried out, everyone explains what the deformity consists of, its characteristics, and etiology, while always clarifying whether a close family member also suffers or has suffered from that deformity. What they also agree on is the treatment, despite being different digital deformities, they all opt for surgery, but one difference between them that should be highlighted is the surgical techniques used. Piette et al., for polydactyly, performs a resection of the second and third rays in addition to the reconstruction of the intermetatarsal ligament. However, Amouzou et al. opts to perform a resection of the medial supernumerary toes, that is, the resection of the first, second, and third toe [10,20].

Elkoun et al., in cases of clinodactyly, recommends performing a middle phalangectomy where the middle phalanx is removed [2,19]. The technique used by Cortés Gómez (2013) in cases of macrodactyly consisted of a “V” incision—both dorsally and planarly—and removing the entire segment, that is, a ray amputation [16].

### 4.2. Bibliographic Studies

A digital deformity that everyone explains is hallux valgus, also popularly known as a bunion. Except for Mézel et al., everyone agrees that the first option would be to opt for conservative treatment, delay surgical treatment as much as possible, and use it when conservative treatment has not been sufficient [20,23,24]. When the deformity is a supraduct or infraduct toe, that is, located above or below, respectively, with respect to the rest, the treatment of choice is conservative, using corrective bandages or silicone orthoses. This type of treatment is also presented by Riera Campillo and Montón Álvarez et al. as the most appropriate [3,4]. It is important to note that for claw and hammer toes, the possibility of individualized conservative treatments, such as silicone orthoses for the second toe or plantar supports, should be considered. While these do not reduce deformity, they do improve quality of life [25].

Except for the article by Arroyave del Río IV et al., as well as clinical cases of a certain deformity, both the articles selected for the study and the majority of those used in the theoretical framework do not only talk about digital deformities, but also many other childhood pathologies such as metatarsus adductus, flat foot, and/or clubfoot [6,15,19,20,21,22,23,24,25].

### 4.3. Limitations of the Study

Although the topic we authors discuss is very common in our consultations, the lack of literature on foot deformities and their possible treatments has led to a very small sample size. Most of the studies found refer to adults or to finger injuries.

Another aspect worth highlighting and taking into account is that only 50% of the sample used for this review refer to studies that present treatments for different cases. The lack of studies in this field leads the authors to consider a future field study.

## 5. Conclusions

1. Common digital deformities in pediatric patients are as follows: Clinodactyly, syndactyly, and macrodactyly. The first, second, and third toes are most affected.
2. Treatment differs depending on the deformity studied. In the case of polydactyly and macrodactyly, or amniotic bands, the only option that is applied is surgery, as well as studying the most appropriate age range to perform it. In others, there are different possible treatments, starting with conservative methods such as orthotics, silicone, corrective bandages, and passive stretching, avoiding surgery or lengthening the surgical procedure as much as possible. Treatment is also given if digital deformity affects or does not affect functionality, as there are some, such as syndactyly and brachydactyly, that do not require it.

Delays in detection can also impact treatment.

## Figures and Tables

**Table 1:** Analysis of the sample articles selected for the study.

Author	Year of Publication	Sample	Age Range	Pathology	Affected Fingers	Treatment
Piette N, Zambelli P, N’Dele D [10].	2017	N = 1	14 months	Isolated heptadactyly (Central polydactyly)	Second and third radius	Surgical treatment. Maintaining the intermetatarsal ligament.
Chang P, Rhodes AC [11].	2011	N = 30	From 0 to 15 years old	Clinodactyly, Syndactyly, Overlapping fingers, Polysyndactyly	Second, third, fourth and fifth	
Elkoun D, Ferrari V, Deroussen F, Planç MC, Klein C, Gouron R [19].	2019	N = 15	From 1 to 5 years	Clinodactyly	Second, third, and fourth	Surgical. Middle phalangectomy.
Cortés Gómez J [16].	2013	N = 46	From 6 months to 9 years	Macrodactyly	First, second, and third	Surgical amputation.
Amouzo KS, Kouevi-Koko TE, Malonga-Loukoula ELJ, Bakriga B, Abalus A [20].	2018	N = 1	10 years	Hexadactilia preaxial (Polidactilia preaxial)	The three medial radii (First, second and third)	Surgical. Resection of the three medial rays.

**Table 2:** Analysis of the bibliographic articles selected for the study.

Author	Year	Pathology	Treatment
Riera Campillo M [3].	2019	Hallux valgus, Campodactyly third and fourth toe.	Conservative treatment (corrective bandage, orthosis…). In exceptional cases of hallux valgus, implement surgical treatment, but delay it as much as possible.
Rampal V, Giuliano F [17].	2020	Brachydactyly, Syndactyly, Polydactyly, Clinodactyly (quintus varus, Hallux varus, Hallux valgus), Campodactyly, Macrodactyly, Amniotic band	In all pathologies, surgery is chosen except in quintus varus, campodactyly, and Hallux valgus, which will require surgery if conservative treatment fails. In the case of syndactyly, if it does not affect function, its treatment is observation.
Mézel A, Manouvrier S [21].	2011	Amniotic band	Surgical
Montón Alvarez JL, Cortés Rico O [4].	2014	First Adductus, Polydactyly, Syndactyly, Clinodactyly, Hallux valgus, Quintus varus, Hammer toes	Surgical treatment: polydactyly during the first year; hallux valgus (when growth ends) and in quintus varus. Conservative always first option.
Arroyave del Río I V, Paola Montoya D, Niño Romero ME [22].	2019	Hallux valgus	Start conservatively (modifications in footwear, orthoses…). Avoid surgery at the age of bone growth.

## Data Availability

Not applicable.

## Supplementary Materials

The following supporting information can be downloaded at https://www.mdpi.com/article/10.3390/children12111461/s1: File S1: PRISMA 2020 Main Checklist. File S2: Caspe_ deformidades digitales.
